# Chronic kidney disease in patients infected with human immunodeficiency virus (HIV) in an urban cohort

**DOI:** 10.1371/journal.pone.0215575

**Published:** 2019-04-17

**Authors:** Rosbel M. Brito, Duc T. Nguyen, Justine R. Johnson, Eric J. Lai, Rochelle E. Castro, Angelina M. Albert, Ann. S. Barnes, Edward A. Graviss, Wadi N. Suki

**Affiliations:** 1 Office of Graduate Medical Education, Houston Methodist Research Institute, Houston, Texas, United States of America; 2 Houston Methodist Research Institute, Houston, Texas, United States of America; 3 Department of Obstetrics and Gynecology, Houston Methodist Hospital, Houston, Texas, United States of America; 4 Nephrology Fellowship Training Program, Houston Methodist Hospital, Houston, Texas, United States of America; 5 Legacy Community Healthcare Center, Houston, Texas, United States of America; 6 Community Education at Methodist Hospital, Houston, Texas, United States of America; 7 Nephrology Training Program at Houston Methodist Hospital, Houston, Texas, United States of America; University of Cape Town, SOUTH AFRICA

## Abstract

**Background and objectives:**

HIV-infected patients are at risk for developing chronic kidney disease (CKD), defined by estimated glomerular filtration rate (eGFR) <60 ml/min/1.73m^2^. Our purpose was to understand the genesis of CKD in HIV patients from a large urban clinic in Houston, Texas, USA, and to characterize progression of CKD in the cohort.

**Design, setting, participants and measurements:**

A retrospective cohort study (2012–2016) was conducted in all HIV-infected patients seen in a federally qualified community health center in Houston, Texas. CKD prevalence and its association with HIV viral load and CD4 count were determined. The association of the change in eGFR over time and comorbidities was assessed using linear mixed models.

**Results:**

Of 3714 HIV-infected patients analyzed, 153 (4.1%) had CKD. The prevalence of CKD in the different racial groups was 5.4% White, 4.0% African American, 2.8% Hispanic/Latino and 3.2% Asian. There was no difference in the rate of decline in kidney function in White vs. African American HIV infected patients with CKD. Compared with non-CKD patients, CKD patients were older, had lived longer with HIV infection, had lower CD4 cell counts, higher proportions of hypertension, hyperlipidemia, and cerebrovascular disease, and had significantly higher rates of eGFR deterioration represented by a median decrease of 26.5% from first to last follow-up eGFR (versus 0% change). Linear mixed modeling identified older age, male gender, White race, longer time with HIV infection, hypertension, history of kidney stones, cerebrovascular disease, autoimmune disease, increased potassium and total cholesterol levels, and being treated with combination ART as associated with a worsening eGFR over time.

**Conclusion:**

This study demonstrates a prevalence of CKD in HIV-infected patients of 4.1% and points to an important role for HIV medications and other common comorbidities in the genesis and progression of kidney disease. Importantly, CKD was not more prevalent in African Americans than in Whites, perhaps due to a low prevalence of IV drug abuse as inferred from the lower prevalence of HCV infection in this cohort.

## Introduction

Renal disease was first reported in HIV-seropositive individuals in 1984 by Rao and colleagues, in a group of ten patients with advanced acquired immune deficiency syndrome (AIDS), and the syndrome was initially designated AIDS nephropathy [1]. The term was later changed to HIV-associated nephropathy (HIVAN). This entity classically presents with heavy proteinuria and rapidly progressive renal failure, and frequently, (although higher CD4 levels in these patients have been reported [2]), an association with low CD4 cell counts and high viral loads [3]. The treatment for HIV infection involves the use of Highly Active Antiretroviral Therapy (HAART) which has been widely accepted since 1996. Typically HAART consists of a combination of two reverse transcriptase inhibitors (RTIs) and a protease inhibitor (PI) or the combination of three RTIs. Reverse transcriptase inhibitors are composed of three classes of inhibitors: Nucleoside RT inhibitors (NRTIs), nucleotide RT inhibitors (NtRTIs), and non-nucleoside RT inhibitors (NNRTIs) [4].

Protease inhibitors (PI) prevent proteolytic cleavage of the HIV Gag and Pol polyproteins that include essential structural and enzymatic components of the virus. Other drugs and combinations are also used including: Integrase Inhibitors, which block HIV integrase enzyme preventing HIV from replicating; entry inhibitors, that block the attachment of HIV gp120 to either the CD4 T cell receptor or the CCR5/CXCR4; and pharmokinetic enhancers that inhibit cytochrome P450 (CYP) 3A enzymes [4].

The kidney plays a significant role in the metabolism and excretion of some antiretroviral (ARV) medications and this causes the kidney to be more susceptible to various types of injuries, including acute renal failure, tubular dysfunction, nephrolithiasis, obstructive nephropathy, and interstitial nephritis [5]. Abnormal renal function has been identified in about 30% of HIV infected patients [6]. Progressive loss in kidney function over a period of months or years, has become one of the leading causes of mortality among patients with HIV infection, and is often associated with other comorbidities that affect kidney function, such as diabetes mellitus and hypertension [7]. Chronic kidney disease (CKD) defined as an estimated glomerular filtration rate (eGFR) of <60 ml/min/1.73 m^2^ [8] is a critical comorbidity for patients infected with HIV, with a reported prevalence between 2.4% and 17% [9]. A systematic review and a meta-analysis done by Ekrikpo et al., reported that the prevalence of CKD in HIV-infected patients varies between geographic regions with a range of 2% to 38%. [10]

While HIVAN is almost exclusively seen in African Americans, and while CKD of any cause is far more common in African Americans than in Americans of European descent, data on CKD not due to HIVAN in HIV infected African Americans is lacking [11].

Currently, the risk of renal disease in patients with HIV infection appears to be compounded by ethnicity, chronic comorbidities, concurrent viral infections, and the use of antiretroviral drugs [12]. Recognition of these chronic comorbidities, co-infections, and anti-retroviral drugs is therefore crucial to the employment of measures that can prevent and or slow the progression to end-stage renal disease (ESRD) [6].

The objectives of the current study were: 1) to determine the prevalence of CKD in a large southwestern urban HIV population, and 2) to identify the relationship between the CKD and HIV viral load, CD4 cell count and comorbidities (such as hypertension and diabetes), and 3) to evaluate the progression of HIV- related CKD.

## Materials and methods

### Study population and design

All adult (aged 19–70) HIV-infected patients seen at a federally qualified community health center (henceforth referred to as the Center) in Houston, Texas during the study period were included in the analysis. A retrospective chart review was undertaken using the electronic medical records from a cohort of HIV-infected patients followed in the Center from January 1, 2012 through October 15, 2016. The population served by this center is predominantly low-income, Hispanic and African-American. The study was approved by the Institutional Review Boards of both the Houston Methodist Hospital and by the CEO of the Center where the study was conducted.

De-identified data was obtained through the i2iTracks Population Health Management (Franklin, Tennessee) from the electronic medical records capturing patients diagnosed with HIV infection who had at least one serum creatinine measurement. Collected data was stored in a REDCap database (Vanderbilt, Tennessee) at Houston Methodist Hospital’s secured data center. Data included demographics, comorbidities, basic and comprehensive metabolic profiles, complete blood count, urinalyses, lipid panel, CD4 count and HIV viral loads, and ART medications.

Glomerular filtration rate (GFR) was estimated from the serum creatinine using the CKD Epidemiology Collaborative Study Equation [13]. Kidney function stage was based on the KDIGO Clinical Practice Guideline [14]. CKD designation was based on the last available serum creatinine and defined by estimated GFR (eGFR) <60 ml/min/1.73m^2^. Change in kidney function in each of the two groups (CKD versus non-CKD) was assessed by the rate of change of eGFR. Consecutive serum creatinine measurements at 48 hrs. or 7-day intervals were not available to exclude acute kidney injury (AKI) as an outcome. Serum creatinine was measured by isotope dilution mass spectrometry (IDMS) traceable technology.

### Statistical methods

Demographic and comorbidity data were reported as median and interquartile range (IQR) for continuous variables, and as frequencies and proportions for categorical variables. Kidney function staging was categorized based on the KDIGO Clinical Practice Guidelines [14]. Differences across groups were compared using Chi-square or Fisher's exact tests for categorical variables and the Kruskal Wallis test for continuous variables as appropriate. The normality of the eGFR distribution was also confirmed using a histogram. Scatter plots with the linear regression line and confidence intervals were used to present the correlations between HIV viral load (with log base 10 transformation) and CD cell count values and the eGFR. Correlations between the eGFR versus log10 HIV viral load and eGFR versus CD4 cell count were taken at the same time point and were determined by the Spearman’s correlation test with a Bonferroni adjustment for multiple measurements. Logistic regression was used to identify the risk factors for CKD. Variables for multivariate models were selected using the Bayesian model averaging (BMA) method [15, 16]. The BMA program suggested good models which included the variables with a high probability of being a risk factor. Variables deemed as clinically important were also evaluated in the multivariate analysis. The Likelihood Ratio test was used to further reduce the model subsets. Model discrimination was determined by the area under the receiver operating characteristic (ROC) curve (AUC). The best model was selected based on the smallest Bayesian information criterion (BIC) and largest AUC. The model's good calibration was determined by a non-significant Hosmer-Lemeshow's goodness of fit test [17]. Potential confounding and effect modification terms were also evaluated. Linear mixed models were also used to assess the association of the change in eGFR over time and potential comorbidities [18]. Post-hoc marginal pairwise comparisons for mean change over time of the eGFR in the entire follow-up course and also per year between the CKD and non-CKD groups were conducted. All analyses were performed on Stata version 15.1 (StataCorp LP, College Station, TX, USA). A p-value of <0.05 was considered statistically significant.

## Results

**Fig 1:**
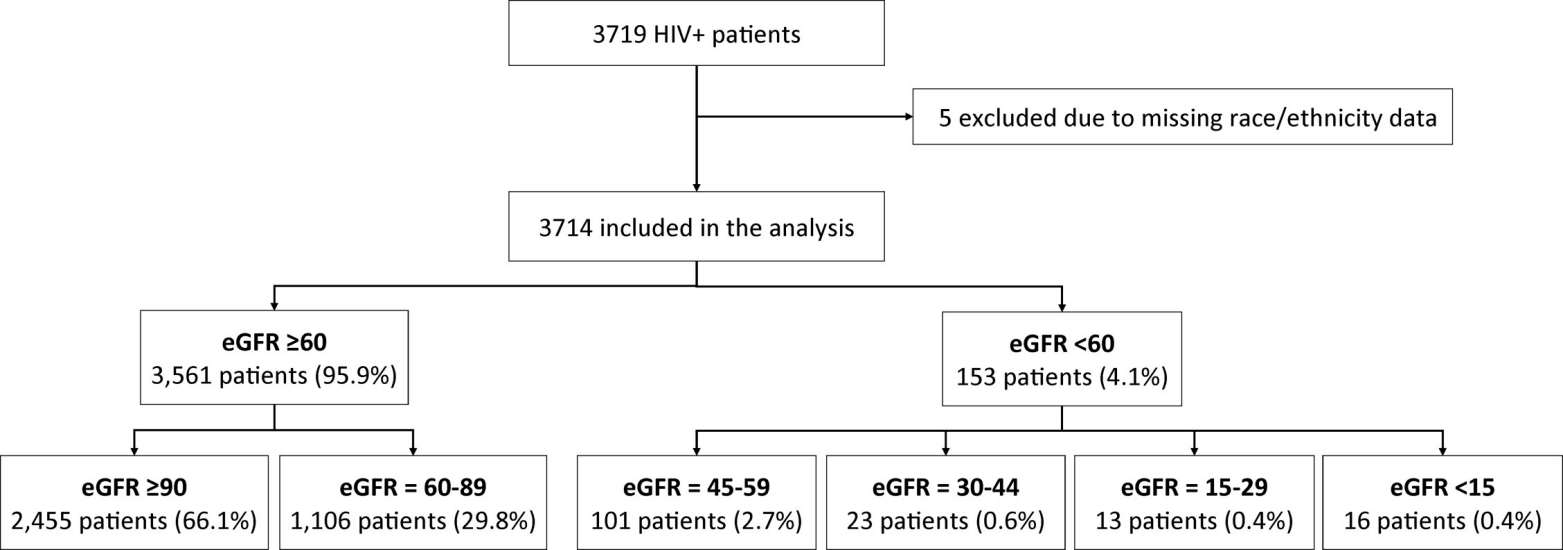
Study flowchart to establish cohort of patients with and without CKD. eGFR unit: ml/min/1.73m^2^.

Of 3719 HIV-infected patients seen at the study center from January 1, 2012 to October 15, 2016, five patients were excluded due to unavailable race/ethnicity. Among the remaining 3,714 patients included in the analysis, 153 (4.1%) had an eGFR<60 ml/min/1.73m^2^ (CKD) and 3,561 (95.9%) had an eGFR ≥60 ml/min/1.73m^2^ (non-CKD) (Fig 1). The patients’ demographics, comorbidities and laboratory parameters are presented in Table 1. Compared with non-CKD patients, patients with CKD were significantly older reflecting their longer time living with HIV infection (Table 1). Smoking, hypertension, hyperlipidemia, and HSV infection were the most frequent comorbidities documented (47.5%, 28.5%, 23.8% and 16.0%, respectively). Other comorbidities (such as peripheral vascular disease, chronic hepatitis C, and tuberculosis) were found in less than 10% of the patients. Hypertension was the most common comorbidity found in the CKD sub-group, which was present in nearly two-third of the patients. Diabetes, while it occurred in only 8.4% of the total cohort, was significantly more prevalent in the CKD vs non-CKD cohort (15% vs. 8.1%; p = 0.003). No significant difference was found in the proportion of smokers between non-CKD and CKD sub-groups (Table 1). CKD patients were more likely to have lower levels of hemoglobin, platelets and albumin and higher levels of total cholesterol and triglycerides than non-CKD patients (Table 1).

**Table 1:** Demographics, comorbidities and laboratory parameters, stratified by CKD.

Variable	Total	eGFR ≥60	eGFR <60	Unadjusted OR	Unadjusted
	(N = 3714)	(*n* = 3561)	(*n* = 153)	(95% CI)	p-value
Age (years), median (IQR)	42.0 (32.0, 51.0)	42.0 (32.0, 51.0)	55.0 (48.0, 60.0)	1.11 (1.09, 1.12)	<0.001
Female gender, n (%)	649 (17.5)	615 (17.3)	34 (22.2)	1.37 (0.93, 2.02)	0.12
Race/Ethnicity, n (%)					
White	925 (24.9)	875 (24.6)	50 (32.7)	1.36 (0.93, 1.99)	0.12
Black	1,511 (40.7)	1,450 (40.7)	61 (39.9)	(reference)	
Hispanic or Latino	1,087 (29.3)	1,056 (29.7)	31 (20.3)	0.70 (0.45, 1.08)	0.11
Asian	62 (1.7)	60 (1.7)	2 (1.3)	0.79 (0.19, 3.32)	0.75
Others	129 (3.5)	120 (3.4)	9 (5.9)	1.78 (0.86, 3.68)	0.12
Years from HIV diagnosis, median (IQR)	4.0 (2.0, 15.0)	4.0 (2.0, 15.0)	13.0 (2.0, 21.0)	1.06 (1.04, 1.08)	<0.001
Hypertension, n (%)	1,057 (28.5)	960 (27.0)	97 (63.4)	4.69 (3.35, 6.57)	<0.001
Diabetes, n (%)	312 (8.4)	289 (8.1)	23 (15.0)	2.00 (1.27, 3.17)	0.003
Hyperlipidemia, n (%)	882 (23.7)	828 (23.3)	54 (35.3)	1.80 (1.28, 2.53)	0.001
History of kidney stone, n (%)	63 (1.7)	56 (1.6)	7 (4.6)	3.00 (1.34, 6.70)	0.01
Smoker, n (%)	1,755 (47.4)	1,693 (47.7)	62 (40.5)	0.75 (0.54, 1.04)	0.08
Cardiovascular disease, n (%)	159 (4.3)	145 (4.1)	14 (9.2)	2.37 (1.34, 4.21)	0.003
Cerebrovascular disease, n (%)	64 (1.7)	54 (1.5)	10 (6.5)	4.54 (2.27, 9.10)	<0.001
Peripheral vascular disease, n (%)	51 (1.4)	48 (1.3)	3 (2.0)	1.46 (0.45, 4.75)	0.53
Chronic HCV, n (%)	350 (9.4)	335 (9.4)	15 (9.8)	1.05 (0.61, 1.80)	0.87
HBV, n (%)	185 (5.0)	171 (4.8)	14 (9.2)	2.00 (1.13, 3.53)	0.02
Tuberculosis, n (%)	69 (1.9)	66 (1.9)	3 (2.0)	1.06 (0.33, 3.41)	0.92
Oral candidiasis, n (%)	321 (8.6)	300 (8.4)	21 (13.7)	1.73 (1.08, 2.78)	0.02
HSV, n (%)	594 (16.0)	562 (15.8)	32 (20.9)	1.41 (0.95, 2.11)	0.09
CMV, n (%)	205 (5.5)	190 (5.3)	15 (9.8)	1.93 (1.11, 3.35)	0.02
WBC (x1000), median (IQR)	5.6 (4.6, 6.9)	5.6 (4.6, 6.9)	5.9 (4.7, 7.2)	1.06 (0.98, 1.15)	0.15
Hemoglobin, median (IQR)	14.4 (13.2, 15.3)	14.4 (13.2, 15.3)	13.5 (11.5, 14.7)	0.74 (0.68, 0.80)	<0.001
Platelets (x1000), median (IQR)	235.5 (201.0, 277.0)	236.0 (201.0, 278.0)	223.0 (183.0, 272.0)	1.00 (0.99, 1.00)	0.004
Potassium, most recent, median (IQR)	4.3 (4.1, 4.6)	4.3 (4.1, 4.5)	4.4 (4.1, 4.7)	1.73 (1.19, 2.51)	0.004
Sodium, most recent, median (IQR)	140.0 (139.0, 141.0)	140.0 (139.0, 141.0)	140.0 (138.0, 141.0)	0.95 (0.89, 1.01)	0.09
Chloride, most recent, median (IQR)	101.0 (99.0, 103.0)	101.0 (99.0, 103.0)	101.0 (99.0, 103.0)	0.97 (0.92, 1.03)	0.32
CO2, most recent, median (IQR)	23.0 (22.0, 25.0)	23.0 (22.0, 25.0)	22.0 (21.0, 25.0)	0.83 (0.78, 0.89)	<0.001
Protein, most recent, median (IQR)	7.3 (7.0, 7.7)	7.3 (7.0, 7.7)	7.3 (6.9, 7.8)	1.08 (0.85, 1.39)	0.53
Albumin, most recent, median (IQR)	4.4 (4.2, 4.6)	4.4 (4.2, 4.6)	4.3 (3.9, 4.5)	0.34 (0.24, 0.49)	<0.001
LDL (x10mg/dL), most recent, median (IQR)	9.2 (7.3, 11.2)	9.2 (7.3, 11.2)	9.4 (7.2, 11.6)	1.02 (0.97, 1.08)	0.39
HDL (x10mg/dL), most recent, median (IQR)	4.5 (3.7, 5.5)	4.5 (3.7, 5.5)	4.7 (3.6, 5.7)	1.09 (0.99, 1.19)	0.08
Total cholesterol (x10mg/dL), most recent, median (IQR)	16.8 (14.5, 19.3)	16.8 (14.5, 19.3)	18.1 (15.1, 20.4)	1.08 (1.04, 1.12)	<0.001
Triglycerides (x10mg/dL), most recent, median (IQR)	12.4 (8.8, 18.3)	12.3 (8.7, 18.2)	15.2 (10.3, 22.0)	1.02 (1.01, 1.03)	<0.001
HIV viral load (log10 copies/mL), median (IQR)	0.0 (0.0, 4.2)	0.0 (0.0, 4.2)	0.0 (0.0, 3.9)	0.98 (0.94, 1.03)	0.46
HIV viral load (copies/mL), n (%)					
<20	2,386 (64.2)	2,282 (64.1)	104 (68.0)	(reference)	
20–99	472 (12.7)	454 (12.7)	18 (11.8)	0.87 (0.52, 1.45)	0.59
100–999	260 (7.0)	251 (7.0)	9 (5.9)	0.79 (0.39, 1.57)	0.50
100–9999	180 (4.8)	175 (4.9)	5 (3.3)	0.63 (0.25, 1.56)	0.32
10000–99999	294 (7.9)	279 (7.8)	15 (9.8)	1.18 (0.68, 2.06)	0.56
≥100000	122 (3.3)	120 (3.4)	2 (1.3)	0.37 (0.09, 1.50)	0.16
HIV viral load ≥50 (copies/mL), n (%)	1,028 (27.7)	989 (27.8)	39 (25.5)	0.89 (0.61, 1.29)	0.54
CD4 cell count (cells/mm3), median (IQR)	582.0 (383.0, 790.0)	587.0 (387.0, 794.0)	488.0 (299.0, 705.0)	0.999 (0.998, 1.00)	0.001
CD4 cell count (log10 cells/mm3), median (IQR)	6.4 (5.9, 6.7)	6.4 (6.0, 6.7)	6.2 (5.7, 6.6)	0.74 (0.61, 0.89)	0.001
CD4 cell count ≤200 (cells/mm3), n (%)	288 (7.8)	271 (7.6)	17 (11.1)	1.52 (0.90, 2.55)	0.12

CKD, Chronic kidney disease (defined by eGFR <60 ml/min/1.73m2); difference across groups was compared using Chi-square test or Fisher’s exact tests for categorical variables and Kruskal Wallis test for continuous variables.

Table 2 presents the results of the multiple logistic regression analysis. The variables suggested for the final model using the BMA algorithm included age, hypertension, cerebrovascular disease, potassium, albumin, cholesterol, triglycerides, integrase inhibitors, and Non-NRTI. HIV viral load and CD4 cell count were also included in the model as they were the main variables of evaluation in this study. Although diabetes and hepatitis C are two clinically important variables, they were not selected by the BMA algorithm. An evaluation of the interaction terms did not find any significant interaction between diabetes, hepatitis C and other covariates. An evaluation on whether diabetes and hepatitis C were confounded by other covariates suggested that these two important comorbidities were most likely confounded by the covariate age. While the OR of diabetes alone in the association with CKD was 2.00 (95% CI 1.27, 3.17; p<0.001), this OR became 0.95 (95% CI 0.59, 1.53; p = 0.83) when adjusted for by age. Given the OR for diabetes after adjusting for age was outside the 95% CI range of the OR of diabetes alone and also in the opposite direction, the association between diabetes and CKD appeared to be confounded by age. In fact, when stratification by age <50 versus ≥50 years occurred, the association between diabetes and CKD became insignificant. In the analysis of the association between hepatitis C and CKD, we also found a similar result with an OR for hepatitis C alone being 1.05 (95% CI 0.61, 1.81, p = 0.87) and 0.60 (95% CI 0.34, 1.04, p = 0.07) when adjusted by age. Because of the confounding of diabetes and hepatitis C with age, these two covariates were not included in the final model.

**Table 2:** Characteristics associated with CKD, multiple logistic regression.

Variable	Adjusted OR (95% CI)	p-value
Age (years)	1.08 (1.06, 1.10)	<0.001
Hypertension	2.34 (1.62, 3.38)	<0.001
History of kidney stone	2.01 (0.95, 4.26)	0.07
Cerebrovascular disease	1.71 (1.14, 2.57)	0.01
Potassium, most recent	0.46 (0.30, 0.70)	<0.001
Albumin	1.05 (1.01, 1.10)	0.02
Total cholesterol (x10mg/dL)	1.01 (1.00, 1.02)	0.13
Triglycerides (x10mg/dL)	1.21 (0.79, 1.87)	0.38
CD4 cell count ≥ 200 (cells/mm3)	1.25 (0.68, 2.31)	0.48
Multiclass Single-Tablet Regimens	1.55 (1.08, 2.24)	0.02
Integrase inhibitors	2.31 (1.53, 3.48)	<0.001
Non NRTI	1.08 (1.06, 1.10)	<0.001

Adjusted OR obtained from the multiple logistic regression model; C statistic = 0.84

### Kidney disease progression

Longitudinal serum creatinine values, which were available in 3714 (96%) patients, were used in the linear mixed modeling to identify prognostic factors associated with the change over time in the individuals’ eGFR. Patients with older age, male gender, White race, longer time with HIV infection, hypertension, history of kidney stone, cerebrovascular disease, autoimmune disease, higher potassium level, higher total cholesterol level, being treated with combined ART were associated with a worse eGFR over time (Table 3).

**Table 3:** Linear mixed model—Characteristics associated with the change in eGFR over the entire course of follow-up.

Characteristics	Coefficient (95% CI)	p-value
Age ≥60 (years)	-12.32 (-17.84, -6.80)	<0.001
Male gender	-0.58 (-3.59, 2.44)	0.71
White	-9.43 (-11.62, -7.23)	<0.001
Years from HIV diagnosis	0.41 (0.27, 0.54)	<0.001
Hypertension	-10.06 (-12.84, -7.27)	<0.001
History of kidney stone	-7.55 (-18.86, 3.75)	0.19
Smoker	0.99 (-0.09, 2.06)	0.07
Cerebrovascular disease	-4.11 (-11.66, 3.43)	0.29
Autoimmune disease	-8.15 (-23.59, 7.29)	0.30
Platelets (x1000)	0.02 (0.01, 0.04)	0.003
Potassium, most recent	-6.79 (-10.23, -3.36)	<0.001
Total cholesterol (x10mg/dL)	-0.59 (-0.88, -0.29)	<0.001
Triglycerides (x10mg/dL)	-0.13 (-0.26, -0.01)	0.04
HIV viral load (log10 copies/mL)	1.54 (0.63, 2.46)	0.001
CD4 cell count (log10 cells/mL)	2.23 (-0.97, 5.43)	0.17
Number of ART groups used		
0		
1	-4.99 (-9.09, -0.89)	0.02
2	-6.14 (-10.39, -1.88)	0.01
3	-4.38 (-8.94, 0.19)	0.06
≥4	-7.90 (-13.63, -2.17)	0.01

Post-hoc marginal pairwise comparison based on the linear mixed model also indicated that CKD patients had a faster decrease in eGFR over time during the full follow-up period compared with non-CKD patients with a mean difference between-group of -45.4 ml/min/1.73m^2^ (95% CI -48.2, -42.6; p<0.001). CKD patients also had a significantly higher rate of eGFR deterioration per year than the non-CKD patients with a median percent eGFR decline per year of -5.8% (-11.2, -2.4) versus -0.2% (-3.2, 2.1), respectively, p<0.001. The rate of decline of eGFR in White patients with CKD was -4.8% (-8.5, -1.2) and -6.7% (-15.0, -2.7) per year in African American patients with CKD (p = 0.21).

**Fig 2:**
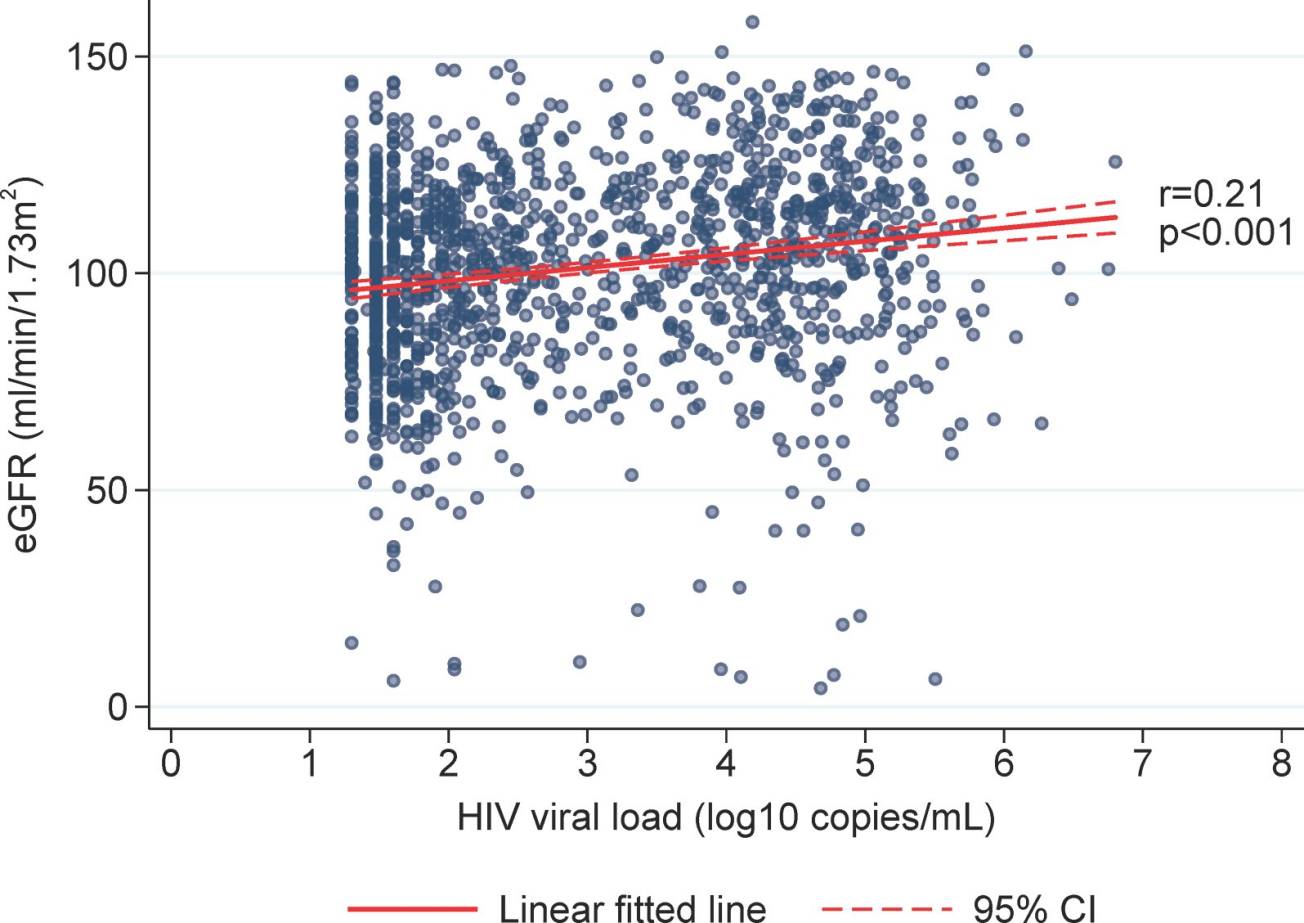
Correlation, eGFR and log10 HIV viral load. Spearman’s correlation test with Bonferroni adjustment for multiple measurements.

**Fig 3:**
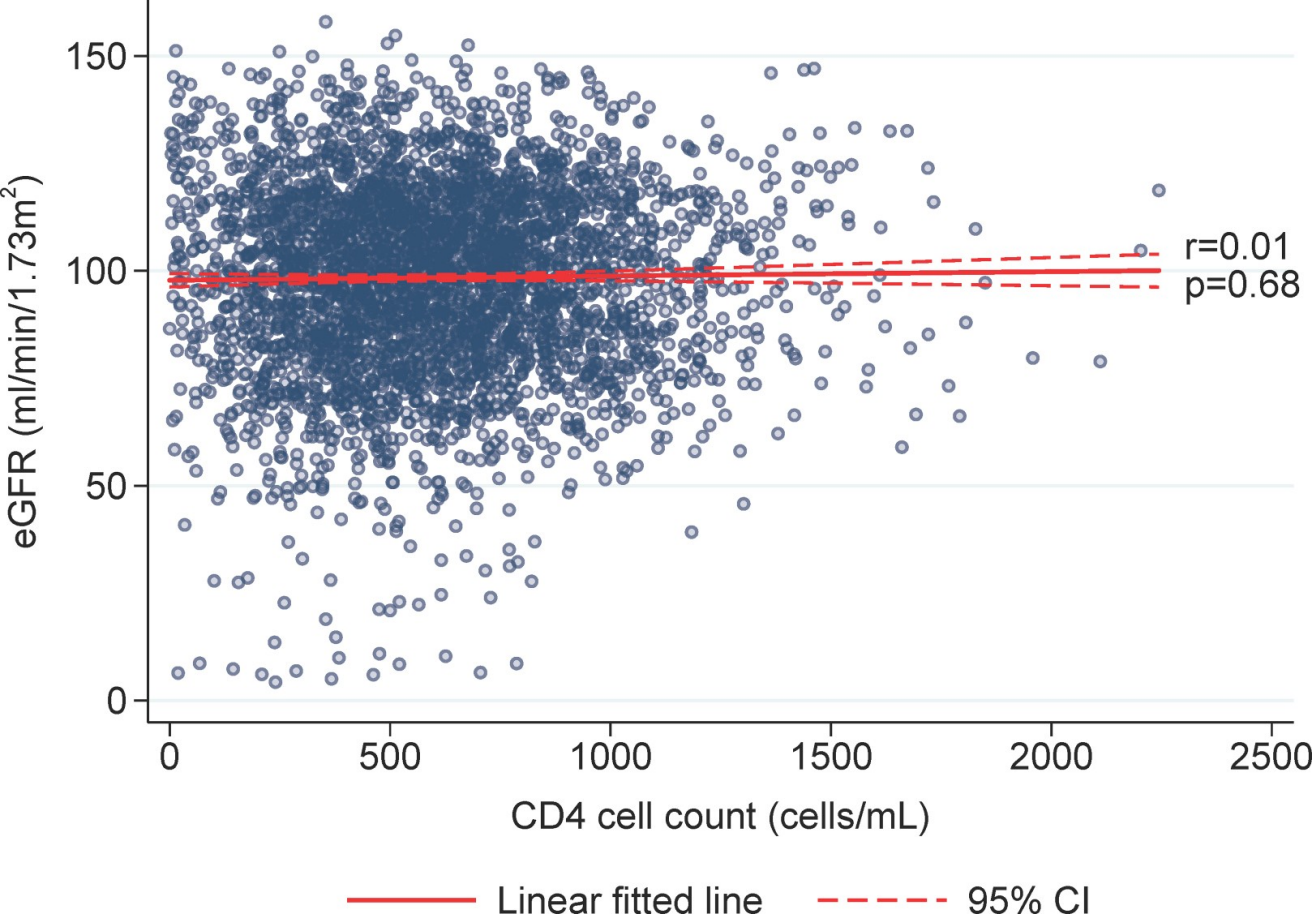
Correlation, eGFR and CD4 count. Spearman’s correlation test with Bonferroni adjustment for multiple measurements.

Spearman’s correlation test with Bonferroni adjustment for multiple measurements suggested that eGFR was significantly correlated with log10 HIV viral load (r = 0.21, p<0.001, Fig 2), but not with CD4 cell count (r = 0.01, p = 0.68, Fig 3). The association of a higher viral load, with a higher eGFR, or conversely a lower eGFR with a lower viral load, could be explained by the adverse renal effects of ART. This is supported by the finding in the linear mixed model analysis of an association between the numbers of ART drugs and worsening of eGFR.

Frequencies of the ART medication groups used in our patients are presented in the Table 4. Most of the patients on ART were treated with combination therapy, the most common of which were: Nucleoside reverse transcriptase inhibitors (NRTI) in 61% of patients; multiclass single tablet regimens NNRTI and two NRTI in 48%; integrase inhibitors and two NRTI in 45%; protease inhibitors and integrase inhibitors in 22%. Among the NRTI medications, emtricitabine/tenofovir was the most commonly prescribed (45%). The most common multiclass single tablet regimens were efavirenz/ emtricitabine /tenofovir (17%), elvitegravir/cobicistat/ emtricitabine/tenofovir (15%) and abacavir/ dolutegravir/lamivudine (14%). Regarding protease inhibitors, ritonavir (36%) and darunavir (26%) were frequently prescribed. Dolutegravir (15%) was the most commonly-used medication among the integrase inhibitors. In 2251 patients who were treated with NRTI medications, 1883 (84%) were treated with tenofovir and 368 (16%) were treated with other NRTIs. Subgroup analysis of the absolute change of eGFR over time in patients treated with NRTI medications indicated that compared with patients treated with other NRTIs, patients treated with tenofovir (TDF) had a statistically significant faster decline of eGFR per year with a median percent change of -0.2% (IQR -0.32, 1.9) versus 0.1% (IQR -2.3, 2.3), p = 0.02. Although statistically significant, this difference is of no clinical significance.

**Table 4:** HIV antiviral therapy in association with CKD.

Variable	Total	eGFR ≥60	eGFR <60	Unadjusted OR	p-value*	
	(N = 3714)	(*n* = 3561)	(*n* = 153)	(95 CI)		
**Number of medications groups used, n (%)**						
0	113 (3.0)	110 (3.1)	3 (2.0)	(reference)		
1	1329 (35.8)	1308 (36.7)	21 (13.7)	0.59 (0.17, 2.00)	0.40	
2	1394 (37.5)	1330 (37.3)	64 (41.8)	1.76 (0.55, 5.71)	0.34	
3	703 (18.9)	657 (18.4)	46 (30.1)	2.57 (0.78, 8.40)	0.12	
4 & 5	175 (4.7)	156 (4.4)	19 (12.4)	4.47 (1.29, 15.46)	0.02	
**Individual medication**						
**Protease Inhibitors, n (%)**						
No	2033 (54.7)	1971 (55.3)	62 (40.5)	(reference)		
Yes	1681 (45.3)	1590 (44.7)	91 (59.5)	1.82 (1.31, 2.53)	<0.001	
**Integrase Inhibitors, n (%)**						
No	2885 (77.7)	2792 (78.4)	93 (60.8)	(reference)		
Yes	829 (22.3)	769 (21.6)	60 (39.2)	2.34 (1.68, 3.27)	<0.001	
**Entry Inhibitors, n (%)**						
No	3677 (99.0)	3529 (99.1)	148 (96.7)	(reference)		
Yes	37 (1.0)	32 (0.9)	5 (3.3)	3.73 (1.43, 9.70)	0.01	
**Pharmacokinetic Enhancer, n (%)**						
No	3713 (100.0)	3560 (100.0)	153 (100.0)			
Yes	1 (<1)	1 (<1)	0 (0.0)	NA		
**Multiclass Single-Tablet Regimens, n (%)**						
No	1918 (51.6)	1810 (50.8)	108 (70.6)	(reference)		
Yes	1796 (48.4)	1751 (49.2)	45 (29.4)	0.43 (0.30, 0.61)	<0.001	
**Non-NRTI, n (%)**						
No	3368 (90.7)	3256 (91.4)	112 (73.2)	(reference)		
Yes	346 (9.3)	305 (8.6)	41 (26.8)	3.91 (2.68, 5.69)	<0.001	
**NRTI, n (%)**						
No	1463 (39.4)	1433 (40.2)	30 (19.6)	(reference)		
Yes	2251 (60.6)	2128 (59.8)	123 (80.4)	2.76 (1.84, 4.14)	<0.001	
**Individual medication, monotherapy only**						
**Protease Inhibitors, n (%)**						
No	1322 (99.5)	1301 (99.5)	21 (100.0)	(reference)		
Yes	7 (0.5)	7 (0.5)	0 (0.0)	6.54 (0, 45.73)	1.00	
**Integrase Inhibitors, n (%)**						
No	1327 (99.8)	1306 (99.8)	21 (100.0)	(reference)		
Yes	2 (0.2)	2 (0.2)	0 (0.0)	25.97 (0, 338.36)	1.00	
**Entry Inhibitors, n (%)**						
No	1329 (100.0)	1308 (100.0)	21 (100.0)			
Yes	0 (0)	0 (0)	0 (0)	NA		
**Pharmacokinetic Enhancer, n (%)**						
No	1329 (100.0)	1308 (100.0)	21 (100.0)			
Yes	0 (0)	0 (0)	0 (0)	NA		
**Multiclass Single-Tablet Regimens, n (%)**						
No	74 (5.6)	73 (5.6)	1 (4.8)	(reference)		
Yes	1255 (94.4)	1235 (94.4)	20 (95.2)	1.18 (0.18, 49.66)	1.00	
**Non-NRTI, n (%)**						
No	1328 (99.9)	1307 (99.9)	21 (100.0)	(reference)		
Yes	1 (0.1)	1 (0.1)	0 (0.0)	62.29 (0, 2429)	1.00	
**NRTI, n (%)**						
No	1265 (95.2)	1245 (95.2)	20 (95.2)	(reference)		
Yes	64 (4.8)	63 (4.8)	1 (4.8)	0.99 (0.02, 6.38)	1.00

Values are in n (%) unless otherwise noted.

*Difference across groups was compared using Chi-square test for categorical variables and Kruskal Wallis test for continuous variables. eGFR unit: ml/min/1.73m^2^; CKD, Chronic kidney disease.

## Discussion

This retrospective cohort study in HIV-infected patients seen in a large, urban U.S. community clinic, evaluated patient characteristics: demographics, comorbidities, laboratory parameters and HARRT medications usage. We found that CKD was associated with older age, male gender, longer time of HIV infection, hypertension, history of kidney stone, cerebrovascular disease, autoimmune disease, higher potassium level, higher total cholesterol level, and being treated with combination ART. Of interest and contrary to expectation, the prevalence of CKD in African Americans in this cohort was not greater than that in White members of the cohort. Furthermore, the rate of decline of eGFR in African Americans with CKD was not greater than that in White patients with CKD. Prior studies have reported that African Americans are at increased risk for incident CKD compared with white subjects and that CKD progression to ESRD is more rapid than in the White HIV-infected population [19, 20]. The lower incidence of CKD in AA than in Whites in the present cohort may be related to the manner by which HIV had been contracted (that is by sexual contact vs. IV drug abuse [IVDA]) (see discussion below regarding HCV prevalence in the present cohort). Of note, in the study by Wong et al, the incidence of IV drug abuse as the cause of HIV infection was greater in the patients with CKD than in those without CKD [20].

Low or undetectable HIV viral load is generally indicative of a high degree of HAART medication compliance. Our data on the possible relationship of HAART medication compliance and the development of CKD in HIV infected patients is conflicting. While there does appear to be a small but significant relationship by linear mixed models between a low viral load and change in eGFR over time, the relationship between the viral load and CKD by multiple logistic regression was not significant. Furthermore, CKD was associated with a significantly lower CD4 count which would argue against greater compliance with HAART medications as the genesis of CKD in HIV infected patients. The previously reported association between TDF treatment and more rapid decline in eGFR although seen in the present cohort is not clinically significant [21, 22].

African Americans patients represented the largest proportion (40.7%) of HIV-infected patients in the cohort, followed by Hispanic or Latino (29.3%). However, despite the fact that African American patients have a genetic predisposition to the development of kidney disease [11], the prevalence of CKD in these patients was consonant with their proportion in the cohort at large. Based on a CDC report in 2015, of newly diagnosed HIV infected patients 48% were African Americans and 24% Hispanic/Latinos. [23]. Therefore, the present cohort is consistent with national HIV prevalence data.

The association of CKD with other comorbidities in this cohort reflects the well-recognized impact of these comorbidities and their prevalence in the general US adult population [12]. During our study period (January 2012 to October 2016), 4.1% of the study population had CKD which falls between the previously reported prevalence estimates of 2.4% and 17% [9]. The availability of longitudinal follow-up data in this cohort allowed us to evaluate progression of kidney disease in CKD patients and compare it to that of patients with better preserved kidney function. The CKD cohort had a more rapid decline in eGFR over the whole follow-up period and per annum compared to non-CKD patients. The CKD in these patients can be due to either associated comorbidities such as hypertension, or to HIV viral infection or its treatment with HAART. In addition, HIV-infected patients with Hepatitis C co-infection are at risk of developing Hepatitis C related nephropathy [24]. In this cohort, the prevalence of Hepatitis C virus (HCV) co-infection was low compared with previous cohort reports [25]; HCV infection was present in 350/3714 patients (9.4%) of the entire cohort and in 15/153 patients (9.8%) in the CKD group. It does not appear from this data therefore that HCV contributes significantly to the CKD in this HIV-infected cohort. In a retrospective cohort study done by the Canadian HIV Observational Cohort (CANOC) from 2000 to 2012, 95% of the population (2462 patients) were screened for HCV and it was found in 484 (20%) of the patients screened [25]. The low incidence of HCV in our population compared to the Canadian cohort may reflect differences with respect to the manner in which HIV was acquired such as sexual contact vs illicit drug use.

In our data, we found that patients with CKD were more likely to experience a lower level of hemoglobin, albumin and CO_2_, but higher levels of total cholesterol, triglycerides and potassium than non-CKD patients. This group also had a significant decrease in their eGFR from the first to the last follow-up period compared with the non-CKD group. The significantly higher odds of CKD in patients treated with integrase inhibitors and NNRTIs as well as the more rapid decline in renal function for patients treated with multiple HIV antiviral medications, suggests that the HAART medications in addition to other co-morbidities may contribute to the incidence and progression of kidney disease in HIV infected patients.

The strength of this study is the use of population-based data available on virtually all patients seen at this large, urban, community HIV clinic and it suggests a regional variation in an important comorbidity, namely HCV infection (a surrogate for IVDA) in the development of CKD. Limitations of this analysis include lack of information on changes in types and dosages of HAART medications or dosage adjustment due to a decrease in renal function, and the use of other potential nephrotoxic agents in the treatment of HIV associated infections.

In conclusion, the present study carried out on a large urban underserved population demonstrates a prevalence of CKD in HIV-infected patients of 4.1% and points to an important role for HIV medications in the genesis and progression of kidney disease in addition to other comorbidities common in the US general population. Of interest and contrary to expectation, the prevalence of CKD and its rate of progression in African Americans in this cohort was not greater than that in White members of the cohort.

## Supporting information

S1 Dataset (XLSX)
